# Gabapentin-lactum–chloranilic acid (1/1)

**DOI:** 10.1107/S1600536809053410

**Published:** 2009-12-16

**Authors:** Jerry P. Jasinski, Ray J. Butcher, Q. N. M. Hakim Al-arique, H. S. Yathirajan, B. Narayana

**Affiliations:** a Department of Chemistry, Keene State College, 229 Main Street, Keene, NH 03435-2001, USA; b Department of Chemistry, Howard University, 525 College Street NW, Washington, DC 20059, USA; c Department of Studies in Chemistry, University of Mysore, Manasagangotri, Mysore 570 006, India; d Department of Studies in Chemistry, Mangalore University, Mangalagangotri 574 199, India

## Abstract

In the title compound, C_9_H_15_NO·C_6_H_2_Cl_2_O_4_ [sytematic name: 2-aza­spiro­[4.5]decan-3-one–chloranilic acid (1/1)], the cyclo­hexane ring of the lactam molecule adopts a slightly distorted normal chair conformation and the five-membered 3-aza­spiro ring is in a slightly distorted chair conformation. The dihedral angle between the least-squares planes of the cyclohexane and 3-azaspiro rings is 84.0 (3)°. In the crystal, the chloranilic acid mol­ecule and the gabapentin-lactum mol­ecules are held together by strong inter­molecular N—H⋯O and O—H⋯O hydrogen bonds with two bifurcated O acceptor atoms on the chloranilic acid mol­ecule and one on the gabapentin-lactum mol­ecule, each bonding with an inter- and intra­molecular hydrogen bond. The molecules are linked into chains parallel to (011) and propagating along the *b* axis.

## Related literature

For the neuroprotective properties of gabapentin-lactam and related compounds, see: Lagreze *et al.* (2001 ▶); Henle *et al.* (2006 ▶); Bowery (1993 ▶). For the synthesis and spectroscopic studies of chloranilic acid charge-transfer complexes, see: Al-Attas *et al.* (2009 ▶). For related structures, see: Gotoh *et al.* (2008 ▶); Ibers (2001 ▶); Ishida (2004 ▶); Ishida & Kashino (2000 ▶); Jasinski *et al.* (2009 ▶). For density functional theory (DFT), see: Frisch *et al.* (2004 ▶); Hehre *et al.* (1986 ▶); Schmidt & Polik (2007 ▶).
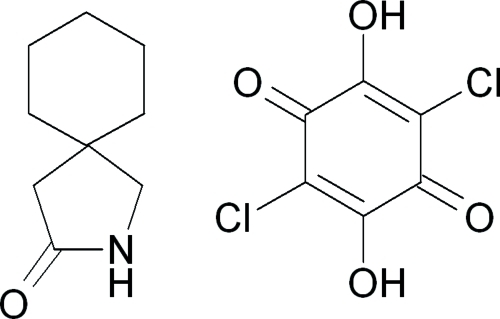


## Experimental

### 

#### Crystal data

C_9_H_15_NO·C_6_H_2_Cl_2_O_4_
*M* *_r_* = 362.20
Triclinic, 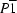

*a* = 6.6127 (9) Å
*b* = 9.5800 (11) Å
*c* = 13.0724 (13) Å
α = 102.679 (9)°
β = 91.934 (9)°
γ = 98.481 (10)°
*V* = 797.23 (16) Å^3^
*Z* = 2
Cu *K*α radiation
μ = 3.90 mm^−1^
*T* = 110 K
0.47 × 0.42 × 0.15 mm

#### Data collection

Goniometer Xcalibur diffractometer with a Ruby (Gemini Cu) detector
Absorption correction: multi-scan (*CrysAlis RED*; Oxford Diffraction, 2007 ▶) *T*_min_ = 0.200, *T*_max_ = 0.557
5138 measured reflections
3123 independent reflections
2731 reflections with *I* > 2σ(*I*)
*R*_int_ = 0.025

#### Refinement

*R*[*F* ^2^ > 2σ(*F* ^2^)] = 0.042
*wR*(*F* ^2^) = 0.119
*S* = 1.05
3123 reflections
210 parameters
H-atom parameters constrained
Δρ_max_ = 0.45 e Å^−3^
Δρ_min_ = −0.40 e Å^−3^

### 

Data collection: *CrysAlis PRO* (Oxford Diffraction, 2007 ▶); cell refinement: *CrysAlis PRO*; data reduction: *CrysAlis PRO*; program(s) used to solve structure: *SHELXS97* (Sheldrick, 2008 ▶); program(s) used to refine structure: *SHELXL97* (Sheldrick, 2008 ▶); molecular graphics: *SHELXTL* (Sheldrick, 2008 ▶); software used to prepare material for publication: *SHELXTL*.

## Supplementary Material

Crystal structure: contains datablocks global, I. DOI: 10.1107/S1600536809053410/ng2704sup1.cif

Structure factors: contains datablocks I. DOI: 10.1107/S1600536809053410/ng2704Isup2.hkl

Additional supplementary materials:  crystallographic information; 3D view; checkCIF report

## Figures and Tables

**Table 1:** Hydrogen-bond geometry (Å, °)

*D*—H⋯*A*	*D*—H	H⋯*A*	*D*⋯*A*	*D*—H⋯*A*
O1*A*—H1*A*⋯O2*A*^i^	0.84	1.97	2.7493 (19)	153
O1*A*—H1*A*⋯O2*A*	0.84	2.20	2.671 (2)	115
O3*A*—H3*A*⋯O1*B*^ii^	0.84	1.70	2.4807 (19)	153
O3*A*—H3*A*⋯O4*A*	0.84	2.26	2.7148 (19)	114
N2*B*—H2*BA*⋯O1*B*^ii^	0.88	2.07	2.913 (2)	161
N2*B*—H2*BA*⋯O4*A*	0.88	2.53	3.091 (2)	122

Symmetry codes: (i) 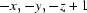; (ii) 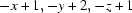.

**Fig. 1.**
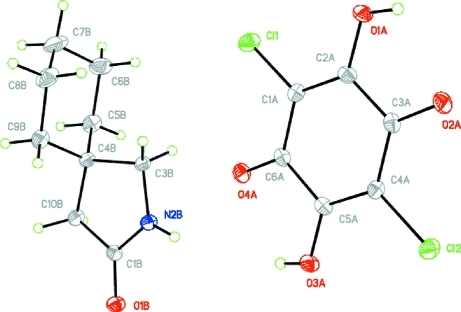
Molecular structure of C9H15NO.C6H2Cl2O4 showing the atom labeling scheme and 50% probability displacement ellipsoids. H atoms are presented as small circles of arbitrary radius.

**Fig. 2.**
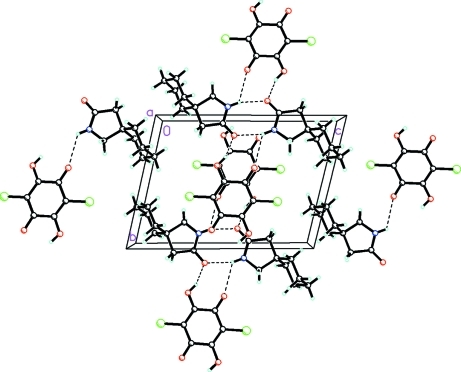
Packing diagram of the title compound, (I), viewed down the a axis. Dashed lines indicate strong N—H···O and O—H···O intermoloecular hydrogen bonds linking the C9H15NO and C6H2Cl2O4 molecules into an infinite O—H···O—H···O—H chain network along the b axis in (011).

## supplementary crystallographic information

### Comment

Gabapentin-lactum (systematic name: 3-azaspiro-[4,5]-decan-2-one), is an intermediate for the preparation of gabapentin. Gabapentin-lactam (GBP-*L*) is also a derivative of the anti-convulsant drug gabapentin. The neuroprotective properties of gabapentin-lactam are described (Lagreze *et al.* 2001). Gabapentin is currently used as a therapeutic agent against epilepsy as well as neuropathic pain. In contrast to gabapentin, its derivative gabapentin-lactam has a pronounced neuroprotective activity (Henle *et al.* 2006). Gabapentin is structurally related to the neurotransmitteraminobutyric acid (GABA), which has been widely studied for its significant inhibitory action in the central nervous system (Bowery, 1993). We have recently reported a crystal structure of a second polymorph of gabapentin hydrochloride hemihydrate with a three-center bifurcated hydrogen bond (Jasinski *et al.* 2009).

Chloranilic acid is a strong dibasic organic acid which exhibits electron-acceptor properties on one hand and acidic properties leading to formation of hydrogen bonds on the other hand. In the case of stronger bases the proton-transfer, hydrogen bonded ion pairs will be formed which is interesting from the point of view of electron transfer reactions in biological systems. Also, protonation of the donor from acidic acceptors are generally a route for the formation of ion pair adducts. The synthesis and spectroscopic studies of charge transfer complexes between chloranilic acid and some heterocyclic amines in ethanol (Al-Attas, Habeeb & Al-Raimi, 2009) have been studied. In view of the importance of gabapentin-lactam, this paper reports the interaction of Gabapentin-lactam as an electron donor with chloranilic acid as an electron acceptor resulting in the formation of a charge transfer complex (I) while the two molecules are held together by intermolecular hydrogen bonding interactions.

The title compound,*C*_9_H_15_NO.C_6_H_2_Cl_2_O_4_,(I), is composed of two independent molecules, gabapentin-lactum (C_9_H_15_NO) and chloranilic acid (C_6_H_2_Cl_2_O_4_),in the asymmetric unit (1:1) (Fig.1). In the gabapentin-lactum molecule the cyclohexane ring (C4B—C9B) adopts a slightly distorted normal chair conformation and the 5-membered 3-Azaspiro ring is in a slightly distorted half-chair conformation The N2B and C1B atoms are *sp*^2^ hybridized while the C3B, C4B and C10B atoms are *sp*^3^. The C10B—C4B—C5B—C6B, N2B—C3B—C4B—C5B and N2B—C3B—C4B—C9B torsion angles are 177.77 (17)°, -136.89 (16)° and 101.19 (17)°, respectively, indicating a significant twist between the 3-azaspiro and cyclohexane rings while sharing a corner C4B atom. The dihedral angle between the least squares planes of these two rings measures 84.0 (3)°. The planar chloranilic acid molecule and gabapentin-lactum molecules are held together by N—H···O and O—H···O intermolecular hydrogen bonds with two bifurcated oxygen acceptor atoms on the chloranilic acid molecule (O2A & O4A) and one on the gabapentin-lactum molecule (O1B), each bifurcating with an inter and intra molecular hydrogen bond, respectively (Fig. 3, Table 1). This produces a set of O—H···O—H···O—H infinite chains along the *b* axis in (011). The O=C—N—H groups from the 3-azaspiro groups in adjacent gabapentin-lactum molecules form a R2,2(8) graph set motif, while the O=C—C—O—H groups from symmetry related chloranilic acid molecules form a R2,2(10) graph set motif in the unit cell (Fig. 3). The dihedral angles between mean planes of the chloranilic acid molecule and the 3-azaspiro and cyclohexane rings of the gabapentin-lactum molecule are 7.0 (1)° and 77.0 (1)°, respectively. In addition, weak *Cg*3···*Cg*3 [= 3.680 (1) Å; slippage = 1.825 Å; -*x*, 1 - *y*,1 - *z*] intermolecular interactions are observed where *Cg*3 = C1A–C6A, which contribute to crystal packing.

Following a geometry optimization, density functional theory (DFT) calculation at the B3LYP 6–31-G(*d*) level (Hehre *et al.*, 1986; Schmidt & Polik, 2007) with the Gaussian03 program package (Frisch *at al.*, 2004) the dihederal angle between the least squares planes of the 3-azaspiro and cyclohexane rings in the gabapentin-lactum molecule become 79.2 (9)° compared to 84.0 (3)° in the crystal. The dihedral angles between mean planes of the chloranilic acid molecule and the 3-azaspiro and cyclohexane rings of the gabapentin-lactum molecule become 2.0 (0)° and 77.2 (9)°, respectively, *versus* 7.0 (1)° and 77.0 (1)° observed in the crystal. Starting geometries were taken from X-ray refinement data. This suggests that strong N—H···O and O—H···O intermolecular hydrogen bonds and weak intermolecular *Cg*···*Cg* intermolecular interactions, collectively, influence crystal packing.

### Experimental

The title compound was synthesized by adding a solution of chloranilic acid (0.42 g, 2 mmol) in 10 ml me thanol to a solution of gabapentin-lactam (0.21 g, 2 mmol) in 10 ml me thanol. A red color developed and the solution was allowed to evaporate slowly at room temperature. The red colored complex formed was filtered off, washed with diethyl ether and dried under vacuum. X-ray quality crystals were grown from methanol:water (80:20 *v*/*v*) solvent mixture (m.p.: 439–442 K). Analysis for C_15_H_17_C_l2_NO_5_: Found (Calculated): C:49.68 (49.74); H: 4.70 (4.73); N:3.85 (3.87).

### Refinement

The hydroxyl and aza hydrogen atoms (H1A, H3A & H2B) were obtained from a difference fourier map. The rest of the H atoms were placed in their calculated positions and then refined using the riding model with O—H = 0.84 Å, N—H = 0.88 Å, C—H = 0.99 Å, and with *U*_iso_(H) = 1.18–1.22*U*_eq_(C,*O*,*N*).

### Crystal data

**Table:**

C_9_H_15_NO·C_6_H_2_Cl_2_O_4_	*Z* = 2
*M**_r_* = 362.20	*F*(000) = 376
Triclinic, *P*1	*D*_x_ = 1.509 Mg m^−^^3^
Hall symbol: -P 1	Cu *K*α radiation, λ = 1.54184 Å
*a* = 6.6127 (9) Å	Cell parameters from 3438 reflections
*b* = 9.5800 (11) Å	θ = 4.8–74.0°
*c* = 13.0724 (13) Å	µ = 3.90 mm^−^^1^
α = 102.679 (9)°	*T* = 110 K
β = 91.934 (9)°	Irregular plate, red-brown
γ = 98.481 (10)°	0.47 × 0.42 × 0.15 mm
*V* = 797.23 (16) Å^3^	

### Data collection

**Table:**

Goniometer Xcalibur diffractometer with a Ruby (Gemini Cu) detector	3123 independent reflections
Radiation source: Enhance (Cu) X-ray Source	2731 reflections with *I* > 2σ(*I*)
graphite	*R*_int_ = 0.025
Detector resolution: 10.5081 pixels mm^-1^	θ_max_ = 74.2°, θ_min_ = 4.8°
ω scans	*h* = −8→5
Absorption correction: multi-scan (*CrysAlis RED*; Oxford Diffraction, 2007)	*k* = −10→11
*T*_min_ = 0.200, *T*_max_ = 0.557	*l* = −15→16
5138 measured reflections	

### Refinement

**Table:**

Refinement on *F*^2^	Primary atom site location: structure-invariant direct methods
Least-squares matrix: full	Secondary atom site location: difference Fourier map
*R*[*F*^2^ > 2σ(*F*^2^)] = 0.042	Hydrogen site location: inferred from neighbouring sites
*wR*(*F*^2^) = 0.119	H-atom parameters constrained
*S* = 1.05	*w* = 1/[σ^2^(*F*_o_^2^) + (0.0762*P*)^2^ + 0.4046*P*] where *P* = (*F*_o_^2^ + 2*F*_c_^2^)/3
3123 reflections	(Δ/σ)_max_ = 0.001
210 parameters	Δρ_max_ = 0.45 e Å^−^^3^
0 restraints	Δρ_min_ = −0.40 e Å^−^^3^

### Special details

**Table:**

Geometry. All e.s.d.'s (except the e.s.d. in the dihedral angle between two l.s. planes) are estimated using the full covariance matrix. The cell e.s.d.'s are taken into account individually in the estimation of e.s.d.'s in distances, angles and torsion angles; correlations between e.s.d.'s in cell parameters are only used when they are defined by crystal symmetry. An approximate (isotropic) treatment of cell e.s.d.'s is used for estimating e.s.d.'s involving l.s. planes.
Refinement. Refinement of *F*^2^ against ALL reflections. The weighted *R*-factor *wR* and goodness of fit *S* are based on *F*^2^, conventional *R*-factors *R* are based on *F*, with *F* set to zero for negative *F*^2^. The threshold expression of *F*^2^ > σ(*F*^2^) is used only for calculating *R*-factors(gt) *etc*. and is not relevant to the choice of reflections for refinement. *R*-factors based on *F*^2^ are statistically about twice as large as those based on *F*, and *R*- factors based on ALL data will be even larger.

### Fractional atomic coordinates and isotropic or equivalent isotropic displacement parameters (Å^2^)

**Table:**

	*x*	*y*	*z*	*U*_iso_*/*U*_eq_	
Cl1	0.07819 (8)	0.35536 (5)	0.25360 (3)	0.02058 (15)	
Cl2	0.30441 (7)	0.39346 (5)	0.73387 (3)	0.01894 (15)	
O1A	0.0035 (2)	0.11592 (14)	0.36708 (11)	0.0185 (3)	
H1A	−0.0101	0.0550	0.4048	0.022*	
O2A	0.1030 (2)	0.13961 (14)	0.57058 (11)	0.0198 (3)	
O3A	0.3781 (2)	0.63101 (14)	0.62265 (11)	0.0168 (3)	
H3A	0.3800	0.6958	0.5885	0.020*	
O4A	0.2633 (2)	0.61192 (14)	0.41806 (11)	0.0174 (3)	
C1A	0.1342 (3)	0.3619 (2)	0.38385 (15)	0.0142 (4)	
C2A	0.0914 (3)	0.2440 (2)	0.42497 (15)	0.0149 (4)	
C3A	0.1454 (3)	0.25105 (19)	0.53885 (15)	0.0142 (4)	
C4A	0.2436 (3)	0.3875 (2)	0.60388 (14)	0.0139 (4)	
C5A	0.2870 (3)	0.50807 (19)	0.56472 (15)	0.0134 (4)	
C6A	0.2301 (3)	0.50147 (19)	0.45017 (15)	0.0132 (4)	
O1B	0.5433 (2)	1.13507 (14)	0.43096 (11)	0.0189 (3)	
C1B	0.4590 (3)	1.03933 (19)	0.35294 (15)	0.0147 (4)	
N2B	0.3806 (3)	0.90640 (16)	0.35775 (12)	0.0158 (3)	
H2BA	0.3923	0.8731	0.4149	0.019*	
C3B	0.2729 (3)	0.8193 (2)	0.26005 (14)	0.0163 (4)	
H3BA	0.1226	0.8081	0.2654	0.020*	
H3BB	0.3141	0.7220	0.2424	0.020*	
C4B	0.3395 (3)	0.90694 (19)	0.17620 (14)	0.0155 (4)	
C5B	0.1561 (3)	0.9111 (2)	0.10248 (16)	0.0214 (4)	
H5BA	0.0461	0.9484	0.1448	0.026*	
H5BB	0.1986	0.9785	0.0568	0.026*	
C6B	0.0727 (4)	0.7610 (2)	0.03391 (17)	0.0288 (5)	
H6BA	0.0190	0.6956	0.0791	0.035*	
H6BB	−0.0418	0.7686	−0.0143	0.035*	
C7B	0.2397 (4)	0.6977 (3)	−0.02991 (17)	0.0320 (5)	
H7BA	0.2827	0.7577	−0.0805	0.038*	
H7BB	0.1841	0.5986	−0.0705	0.038*	
C8B	0.4253 (4)	0.6919 (2)	0.04056 (17)	0.0278 (5)	
H8BA	0.5350	0.6579	−0.0036	0.033*	
H8BB	0.3866	0.6216	0.0847	0.033*	
C9B	0.5062 (3)	0.8409 (2)	0.11141 (16)	0.0198 (4)	
H9BA	0.5625	0.9073	0.0674	0.024*	
H9BB	0.6193	0.8318	0.1598	0.024*	
C10B	0.4297 (3)	1.0593 (2)	0.24256 (15)	0.0175 (4)	
H10A	0.5621	1.0955	0.2170	0.021*	
H10B	0.3340	1.1288	0.2394	0.021*	

### Atomic displacement parameters (Å^2^)

**Table:**

	*U*^11^	*U*^22^	*U*^33^	*U*^12^	*U*^13^	*U*^23^
Cl1	0.0306 (3)	0.0172 (2)	0.0145 (2)	0.00588 (19)	−0.00172 (18)	0.00410 (18)
Cl2	0.0277 (3)	0.0151 (2)	0.0153 (2)	0.00428 (18)	−0.00131 (18)	0.00606 (17)
O1A	0.0265 (7)	0.0099 (6)	0.0176 (7)	−0.0005 (5)	0.0004 (6)	0.0026 (5)
O2A	0.0281 (8)	0.0111 (6)	0.0211 (7)	0.0016 (6)	0.0049 (6)	0.0057 (5)
O3A	0.0228 (7)	0.0093 (6)	0.0179 (7)	−0.0007 (5)	−0.0029 (5)	0.0048 (5)
O4A	0.0214 (7)	0.0131 (7)	0.0194 (7)	0.0022 (5)	0.0020 (5)	0.0076 (5)
C1A	0.0154 (9)	0.0149 (9)	0.0141 (9)	0.0062 (7)	0.0023 (7)	0.0042 (7)
C2A	0.0144 (9)	0.0112 (8)	0.0183 (9)	0.0032 (7)	0.0023 (7)	0.0008 (7)
C3A	0.0154 (9)	0.0107 (9)	0.0184 (9)	0.0048 (7)	0.0048 (7)	0.0047 (7)
C4A	0.0168 (9)	0.0125 (9)	0.0138 (9)	0.0051 (7)	0.0017 (7)	0.0042 (7)
C5A	0.0112 (9)	0.0113 (9)	0.0184 (9)	0.0036 (7)	0.0014 (7)	0.0037 (7)
C6A	0.0122 (8)	0.0108 (9)	0.0179 (9)	0.0043 (7)	0.0027 (7)	0.0044 (7)
O1B	0.0276 (8)	0.0112 (6)	0.0166 (7)	−0.0010 (5)	−0.0015 (6)	0.0036 (5)
C1B	0.0170 (9)	0.0110 (8)	0.0166 (9)	0.0029 (7)	0.0014 (7)	0.0037 (7)
N2B	0.0236 (8)	0.0109 (7)	0.0130 (8)	0.0012 (6)	−0.0001 (6)	0.0044 (6)
C3B	0.0245 (10)	0.0116 (8)	0.0123 (9)	0.0004 (7)	0.0009 (7)	0.0032 (7)
C4B	0.0224 (10)	0.0106 (9)	0.0138 (9)	0.0024 (7)	0.0013 (7)	0.0038 (7)
C5B	0.0251 (10)	0.0216 (10)	0.0192 (10)	0.0078 (8)	−0.0004 (8)	0.0058 (8)
C6B	0.0350 (12)	0.0279 (12)	0.0208 (10)	0.0012 (9)	−0.0075 (9)	0.0034 (9)
C7B	0.0492 (15)	0.0252 (11)	0.0174 (10)	0.0041 (10)	−0.0010 (10)	−0.0022 (8)
C8B	0.0413 (13)	0.0195 (10)	0.0229 (10)	0.0115 (9)	0.0078 (9)	0.0002 (8)
C9B	0.0245 (10)	0.0173 (9)	0.0193 (9)	0.0054 (8)	0.0042 (8)	0.0060 (8)
C10B	0.0268 (10)	0.0112 (9)	0.0155 (9)	0.0030 (7)	0.0024 (8)	0.0049 (7)

### Geometric parameters (Å, °)

**Table:**

Cl1—C1A	1.7158 (19)	C3B—H3BB	0.9900
Cl2—C4A	1.7196 (18)	C4B—C5B	1.534 (3)
O1A—C2A	1.330 (2)	C4B—C9B	1.538 (3)
O1A—H1A	0.8400	C4B—C10B	1.546 (2)
O2A—C3A	1.227 (2)	C5B—C6B	1.530 (3)
O3A—C5A	1.301 (2)	C5B—H5BA	0.9900
O3A—H3A	0.8400	C5B—H5BB	0.9900
O4A—C6A	1.215 (2)	C6B—C7B	1.523 (3)
C1A—C2A	1.352 (3)	C6B—H6BA	0.9900
C1A—C6A	1.465 (3)	C6B—H6BB	0.9900
C2A—C3A	1.504 (3)	C7B—C8B	1.525 (3)
C3A—C4A	1.441 (3)	C7B—H7BA	0.9900
C4A—C5A	1.361 (3)	C7B—H7BB	0.9900
C5A—C6A	1.517 (3)	C8B—C9B	1.531 (3)
O1B—C1B	1.262 (2)	C8B—H8BA	0.9900
C1B—N2B	1.318 (2)	C8B—H8BB	0.9900
C1B—C10B	1.507 (2)	C9B—H9BA	0.9900
N2B—C3B	1.460 (2)	C9B—H9BB	0.9900
N2B—H2BA	0.8800	C10B—H10A	0.9900
C3B—C4B	1.558 (2)	C10B—H10B	0.9900
C3B—H3BA	0.9900		
			
C2A—O1A—H1A	109.5	C10B—C4B—C3B	103.66 (14)
C5A—O3A—H3A	109.5	C6B—C5B—C4B	111.76 (17)
C2A—C1A—C6A	120.62 (17)	C6B—C5B—H5BA	109.3
C2A—C1A—Cl1	121.97 (15)	C4B—C5B—H5BA	109.3
C6A—C1A—Cl1	117.41 (14)	C6B—C5B—H5BB	109.3
O1A—C2A—C1A	122.13 (17)	C4B—C5B—H5BB	109.3
O1A—C2A—C3A	116.63 (16)	H5BA—C5B—H5BB	107.9
C1A—C2A—C3A	121.23 (16)	C7B—C6B—C5B	110.90 (19)
O2A—C3A—C4A	124.02 (17)	C7B—C6B—H6BA	109.5
O2A—C3A—C2A	117.66 (17)	C5B—C6B—H6BA	109.5
C4A—C3A—C2A	118.32 (16)	C7B—C6B—H6BB	109.5
C5A—C4A—C3A	121.67 (17)	C5B—C6B—H6BB	109.5
C5A—C4A—Cl2	120.66 (14)	H6BA—C6B—H6BB	108.0
C3A—C4A—Cl2	117.67 (14)	C6B—C7B—C8B	111.52 (18)
O3A—C5A—C4A	121.97 (17)	C6B—C7B—H7BA	109.3
O3A—C5A—C6A	118.01 (16)	C8B—C7B—H7BA	109.3
C4A—C5A—C6A	120.03 (16)	C6B—C7B—H7BB	109.3
O4A—C6A—C1A	123.11 (17)	C8B—C7B—H7BB	109.3
O4A—C6A—C5A	118.78 (16)	H7BA—C7B—H7BB	108.0
C1A—C6A—C5A	118.10 (15)	C7B—C8B—C9B	111.18 (17)
O1B—C1B—N2B	123.96 (17)	C7B—C8B—H8BA	109.4
O1B—C1B—C10B	125.82 (16)	C9B—C8B—H8BA	109.4
N2B—C1B—C10B	110.21 (16)	C7B—C8B—H8BB	109.4
C1B—N2B—C3B	114.19 (15)	C9B—C8B—H8BB	109.4
C1B—N2B—H2BA	122.9	H8BA—C8B—H8BB	108.0
C3B—N2B—H2BA	122.9	C8B—C9B—C4B	112.62 (17)
N2B—C3B—C4B	104.11 (15)	C8B—C9B—H9BA	109.1
N2B—C3B—H3BA	110.9	C4B—C9B—H9BA	109.1
C4B—C3B—H3BA	110.9	C8B—C9B—H9BB	109.1
N2B—C3B—H3BB	110.9	C4B—C9B—H9BB	109.1
C4B—C3B—H3BB	110.9	H9BA—C9B—H9BB	107.8
H3BA—C3B—H3BB	109.0	C1B—C10B—C4B	105.01 (15)
C5B—C4B—C9B	109.57 (15)	C1B—C10B—H10A	110.7
C5B—C4B—C10B	111.96 (15)	C4B—C10B—H10A	110.7
C9B—C4B—C10B	109.81 (16)	C1B—C10B—H10B	110.7
C5B—C4B—C3B	111.08 (16)	C4B—C10B—H10B	110.7
C9B—C4B—C3B	110.64 (15)	H10A—C10B—H10B	108.8
			
C6A—C1A—C2A—O1A	179.69 (16)	C4A—C5A—C6A—C1A	−1.8 (3)
Cl1—C1A—C2A—O1A	0.0 (3)	O1B—C1B—N2B—C3B	174.06 (18)
C6A—C1A—C2A—C3A	−1.6 (3)	C10B—C1B—N2B—C3B	−5.0 (2)
Cl1—C1A—C2A—C3A	178.76 (13)	C1B—N2B—C3B—C4B	14.0 (2)
O1A—C2A—C3A—O2A	−0.8 (3)	N2B—C3B—C4B—C5B	−136.89 (16)
C1A—C2A—C3A—O2A	−179.58 (18)	N2B—C3B—C4B—C9B	101.19 (17)
O1A—C2A—C3A—C4A	179.11 (16)	N2B—C3B—C4B—C10B	−16.48 (19)
C1A—C2A—C3A—C4A	0.3 (3)	C9B—C4B—C5B—C6B	55.7 (2)
O2A—C3A—C4A—C5A	−179.95 (18)	C10B—C4B—C5B—C6B	177.77 (17)
C2A—C3A—C4A—C5A	0.2 (3)	C3B—C4B—C5B—C6B	−66.9 (2)
O2A—C3A—C4A—Cl2	0.0 (3)	C4B—C5B—C6B—C7B	−57.1 (2)
C2A—C3A—C4A—Cl2	−179.94 (13)	C5B—C6B—C7B—C8B	55.9 (2)
C3A—C4A—C5A—O3A	−179.05 (16)	C6B—C7B—C8B—C9B	−54.4 (3)
Cl2—C4A—C5A—O3A	1.1 (3)	C7B—C8B—C9B—C4B	54.4 (2)
C3A—C4A—C5A—C6A	0.6 (3)	C5B—C4B—C9B—C8B	−54.5 (2)
Cl2—C4A—C5A—C6A	−179.28 (13)	C10B—C4B—C9B—C8B	−177.91 (16)
C2A—C1A—C6A—O4A	−176.63 (18)	C3B—C4B—C9B—C8B	68.3 (2)
Cl1—C1A—C6A—O4A	3.0 (3)	O1B—C1B—C10B—C4B	174.58 (18)
C2A—C1A—C6A—C5A	2.3 (3)	N2B—C1B—C10B—C4B	−6.4 (2)
Cl1—C1A—C6A—C5A	−178.02 (13)	C5B—C4B—C10B—C1B	133.80 (17)
O3A—C5A—C6A—O4A	−3.2 (3)	C9B—C4B—C10B—C1B	−104.25 (17)
C4A—C5A—C6A—O4A	177.16 (17)	C3B—C4B—C10B—C1B	13.99 (19)
O3A—C5A—C6A—C1A	177.86 (15)		

### Hydrogen-bond geometry (Å, °)

**Table:**

*D*—H···*A*	*D*—H	H···*A*	*D*···*A*	*D*—H···*A*
O1A—H1A···O2A^i^	0.84	1.97	2.7493 (19)	153
O1A—H1A···O2A	0.84	2.20	2.671 (2)	115
O3A—H3A···O1B^ii^	0.84	1.70	2.4807 (19)	153
O3A—H3A···O4A	0.84	2.26	2.7148 (19)	114
N2B—H2BA···O1B^ii^	0.88	2.07	2.913 (2)	161
N2B—H2BA···O4A	0.88	2.53	3.091 (2)	122

Symmetry codes: (i) −*x*, −*y*, −*z*+1; (ii) −*x*+1, −*y*+2, −*z*+1.
